# Rumen and Hindgut Bacteria Are Potential Indicators for Mastitis of Mid-Lactating Holstein Dairy Cows

**DOI:** 10.3390/microorganisms8122042

**Published:** 2020-12-20

**Authors:** Yifan Zhong, Ming-Yuan Xue, Hui-Zeng Sun, Teresa G. Valencak, Le Luo Guan, Jianxin Liu

**Affiliations:** 1 Institute of Diary Science, College of Animal Sciences, Zhejiang University, Hangzhou 310058, China; yifanzhong@zju.edu.cn (Y.Z.); myxue@zju.edu.cn (M.-Y.X.); huizeng@zju.edu.cn (H.-Z.S.); teresavalencak@zju.edu.cn (T.G.V.); 2 Department of Agricultural, Food and Nutritional Science, University of Alberta, Edmonton, AB T6G 2P5, Canada; lguan@ualberta.ca

**Keywords:** mastitis, dairy cows, rumen bacteria, hindgut bacteria, random forest, milk production

## Abstract

Mastitis is one of the major problems for the productivity of dairy cows and its classifications have usually been based on milk somatic cell counts (SCCs). In this study, we investigated the differences in milk production, rumen fermentation parameters, and diversity and composition of rumen and hindgut bacteria in cows with similar SCCs with the aim to identify whether they can be potential microbial biomarkers to improve the diagnostics of mastitis. A total of 20 dairy cows with SCCs over 500 × 10^3^ cells/mL in milk but without clinical symptoms of mastitis were selected in this study. Random forest modeling revealed that *Erysipelotrichaceae* UCG 004 and the [*Eubacterium*] *xylanophilum* group in the rumen, as well as the Family XIII AD3011 group and *Bacteroides* in the hindgut, were the most influential candidates as key bacterial markers for differentiating “true” mastitis from cows with high SCCs. Mastitis statuses of 334 dairy cows were evaluated, and 96 in 101 cows with high SCCs were defined as healthy rather than mastitis according to the rumen bacteria. Our findings suggested that bacteria in the rumen and hindgut can be a new approach and provide an opportunity to reduce common errors in the detection of mastitis.

## 1. Introduction

Mastitis is among the most prevalent and costly diseases in dairy cows that is one of the health problems in udders impacting dairy cow productivity and health [1]. Mastitis can usually be classified into clinical and subclinical mastitis, of which the latter is the most common [2] but difficult to detect timely and accurately because of its invisible symptoms in udders [3]. To defend against the pathogen infections, immune cells are recruited in the mammary gland tissue and are released from the tissue to the milk, which leads to the elevation of milk somatic cell count (SCC) [4] as one of the rapid and practical measures to monitor mastitis in dairy cows for decades in the global dairy industry compared with other methods [5]. Although there is a consensus that infection status and an increased SCC are parallel, the optimal threshold of SCC in milk for subclinical mastitis remains variable in different countries [6], suggesting the ambiguity for discrimination of subclinical mastitis with SCC in milk. The false positive usually occurred with the diagnosis of subclinical mastitis based solely on SCC measurement [7], which can be erroneous when solely relying on a single SCC test [8]. Especially when SCCs in the milk are over 500 × 10^3^ cells/mL, the cows are considered as subclinical mastitis, and commonly, these cows are isolated and treated with antibiotics. Mastitis can have a multidirectional impact on animal production, including economic losses, reproductive disorders, etc., and consequently cause challenges to the dairy processing industry [9]. However, in some cases, not all cows are “true” mastitis; in some cases, despite being diagnosed as “subclinical mastitis” according to the milk SCC, it suggests the need to have a more powerful tool to further discriminate the “true” mastitis statuses of dairy cows with the higher SCC in milk.

To minimize the misdiagnosis of animals from the mastitis condition [10] for better prevention and treatment, technological interventions in the diagnosis of cow health in the herd have been proposed [11]. In humans and nonhuman mammals, the suppression and over-colonization of certain bacterial species in the gastrointestinal tract result in increasing disease pathogenicity and emphasize the importance of understanding the interaction between a host and its inhabiting commensal microbes [12]. Thus, knowing the abundance of certain gastrointestinal bacteria can be used for the classification or prediction of the statuses of dairy cows [13,14]. Recently, Hu et al. [15] demonstrated that gut microbiota act as protective factors in the host defense system against mastitis in mice and that the gut–mammary gland axis represents a new and promising therapeutic approach for the treatment of mastitis. Indeed, Ma et al. [16] further confirmed that the transplantation of fecal microbiota from cows affected by mastitis to germ-free mice led to mastitis symptoms, indicating that the dysbiosis of gut bacteria may lead to mastitis. Moreover, our previous studies reported that rumen bacteria differ between high- and low-SCC cows [17]. The studies above indicate that the potential interaction between mastitis and gastrointestinal bacteria in cows may exist, possibly through metabolites or the translocation of certain bacteria by an entero-mammary pathway [18].

As dairy cows may be “true” mastitis (MA) while some of them are mistakenly classified as “subclinical mastitis” (SC) when the milk SCC is employed as the only discrimination of mastitis in dairy farms, we hypothesized that there exists a variation in both rumen and hindgut bacteria between SC and MA cows, which may be predictive markers for “true” mastitis. Therefore, the rumen and hindgut bacteria were profiled in cows with high SCC in this study, aiming to evaluate the predictive capability of microbial markers from both the rumen and hindgut for ”true“ mastitis using a random forest machine-learning algorithm.

## 2. Materials and Methods

### 2.1. Ethics Statement

All animal work and methods used in this study were approved by the Animal Care Committee of Zhejiang University (Hangzhou, China) and were in accordance with the University’s guidelines for animal research.

### 2.2. Experiment Design

In total, 20 Holstein mid-lactation dairy cows (parity = 2.05 ± 0.94, days in milk = 166 ± 24, mean ± SD) and identified as having “subclinical mastitis” (SCC > 500 × 10^3^ cells/mL) were selected for the study from a commercial dairy farm (Hangzhou, China). All cows were kept at the same management conditions when identified as “subclinical mastitis” cases while showing no clinical disease symptoms but high SCC in milk. The cows were fed ad libitum with a total mixed ratio (Table S1) for intake and had free access to clean water. Animals were divided into SC (*n* = 9) and MA (*n* = 11) according to rumen and hindgut bacteria patterns together with the physiology statuses.

### 2.3. Sample Collection and Analysis

On the sampling day, the individual milk yield was recorded, and milk samples were collected for the measurement of milk protein, fat, lactose, urea nitrogen, and SCC by infrared analysis [19] using a Foss FT+ instrument (Foss Electric, Hillerød, Denmark). Rumen fluid was collected by using oral stomach tubes [20] before the morning feeding, and the rumen fluid pH was measured immediately using a pH meter (FE-20-FiveEasy PlusTM; Mettler Toledo Instruments Co. Ltd., Shanghai, China). The rumen samples were stored at −80 °C until further analysis. The ammonia-N concentration was determined using steam distillation into boric acid and titration with dilute hydrochloric acid, and gas chromatography was used for the analysis of volatile fatty acid (VFA) concentrations [21]. Fecal samples were collected from the rectum before feeding in the morning and stored immediately at −80 °C until further analysis.

### 2.4. DNA Extraction and Sequencing

The bead-beating method was used for total DNA extraction from rumen and fecal samples [22]. The DNA quality was measured by a NanoDrop 2000 Spectrophotometer (NanoDrop Technologies, Wilmington, DE, USA). The 341F/806R primer set (338 F: 5′-ACTCCTACGGGAGGCAGCAG-3′, 806R: 5′-GGACTACHVGGGTWTCTAAT-3′) was used to generate amplicons that target the V3–V4 region of the bacterial 16S rRNA gene. The PCR solution (25 μL) contained 0.5 U of Taq polymerase (TransGen Biotech, Beijing, China) in 25 μL of 10 × PCR buffer, 200 μM of each dNTP, 0.2 μM of each primer, and 2 μL of DNA (50 ng/μL). A Phusion High-Fidelity PCR Mastermix (New England Biolabs (Beijing) Ltd., China) was used for PCR reaction with the following program: 94 °C for 3 m; 35 cycles at 94 °C for 45 s, 50 °C for 60 s, and 72 °C for 90 s; and 72 °C for 10 m. The PCR products were visualized on 2% agarose gels, purified using a QIAquick gel extraction kit (Qiagen, Dusseldorf, Germany) and then sequenced on an Illumina MiSeq platform (San Diego, CA, USA) using pair-ended 2 × 300 bp in Majorbio Bioinformatics Technology Co. Ltd. (Shanghai, China). The raw 16S rRNA gene sequences were deposited in the NCBI Sequence Read Archive (PRJNA526913).

### 2.5. Sequence Analyses

QIIME 2 (version 2018.11) was used for demultiplexing and processing of the raw fastq files (https://qiime2.org). Q2-DEMUX (https://github.com/qiime2/q2-demux) was used for the demultiplexing of reads, and the Q2-DADA2 pipeline [23] was used for filtering, dereplication, chimera identification, and merging paired-end reads. The SILVA database (version 132, https://www.arb-silva.de) was used for the taxonomy classification of representative sequences sets. Shannon, Simpson, Ace, and Chao 1 indices were calculated using QIIME2. Beta diversity was evaluated using Bray–Curtis and Weighted UniFrac distances were calculated in QIIME2 and visualized using principal coordinate analysis (PCoA) in R software (version 3.3.1).

### 2.6. Statistical Analyses

For all analyses, the *p* values were adjusted for false discovery rate (FDR) using the Benjamini–Hochberg method, and significance was determined as *p* < 0.05. The performance and rumen fermentation parameters were calculated using Student’s *t*-test. The Kruskal–Wallis test was performed to explore differences in alpha diversities (Shannon, Simpson, Ace, and Chao 1 index) and the relative abundance of rumen and hindgut bacteria between SC and MA cows. Bray–Curtis and weighted dissimilarity matrixes were used to evaluate the belonging to a bacterial community. Principal coordinate analysis (PCoA) was applied to identify the dissimilarity matrixes for visualization.

To find out if the rumen and hindgut microbiome could be used to predict “true” mastitis in dairy cows, random forest modeling (R package “randomForest,” version 4.6-14) was used to identify microbial signatures that accurately differentiated the ”true“ mastitis of dairy cows. All genera with a relative abundance over 0.1% were included as inputs into the random forest model. The machine learning technique accounts for nonlinear relationships and dependencies between all genera. A score reflecting the importance (MDA: Mean decrease accuracy) was given to each genus based on the increase in error caused by removing that genus from the predictors. Random forest modeling uses 70% of the data as a “training” data set by random sampling with replacement and validates the selected genus using the remaining “out-of-bag” samples. We identified the best predictive model based on the maximum area under the curve (AUC) by using the AUC-RF-algorithm.

To validate the predictability of “true” mastitis based on the random forest model constructed, we further used the rumen bacteria dataset obtained from a large cohort in our previous study that consisted of 334 lactating dairy cows [24] who were raised in another farm and had no clinical signs of mastitis. The data and analyses of the rumen bacteria were used in the QIIME2 pipeline, with the procedures as described before [25]. The amplicon sequence variants (ASVs) were assigned based on the SILVA 132 database (https://www.arb-silva.de), and the relative abundances of rumen bacteria and SCC records of 334 dairy cows are shown in Table S2.

## 3. Results

### 3.1. Performance and Rumen Fermentation

As shown in Table 1, both SC and MA individuals had high SCCs in milk, while there were no significant differences in parity and lactation stage. SC cows showed significantly lower milk yield (*p* < 0.01), percentage of lactose (*p* = 0.04), and concentration of milk urea nitrogen (*p* < 0.01) than individuals from the SC group.

The Rumen pH and ruminal concentration of total volatile fatty acids showed no significant differences between the SC and the MA groups (Table 2). Compared to the SC group, a higher molar proportion of acetate (*p* < 0.01) and lower percentages of butyrate (*p* < 0.01), isovalerate (*p* = 0.02), and valerate (*p* = 0.01) were observed in the rumen of the MA group. Besides, the A:P ratio, reflecting the relationship between acetate and propionate, was higher in the MA group than that in the SC group (*p* = 0.01).

### 3.2. Rumen and Hindgut Bacteria Communities

After removing low-quality reads and chimeras using QIIME 2 (2018.11), 393,200 and 422,070 high-quality reads remained for rumen and hindgut samples, respectively (Table S3). These sequences were assigned to 5200 and 2865 features based on the 100% similarity for rumen and hindgut samples. The sequence number was normalized to 19,660 for rumen samples and 9294 for hindgut samples to standardize the sampling for downstream alpha and beta diversity analyses.

**Figure 1:**
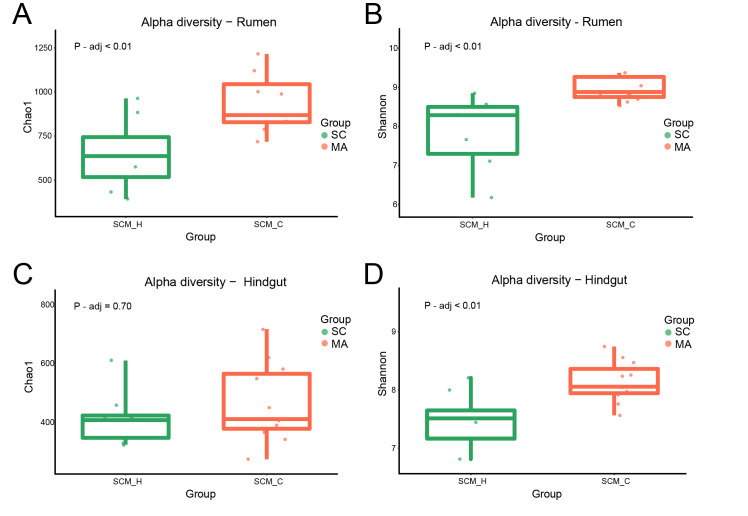
Alpha diversity of rumen and hindgut bacteria between high-somatic-cell-counts cows with healthy patterns (SC) and mastitis patterns (MA). (**A**) Chao 1 index and (**B**) Shannon index of rumen bacteria; (**C**) Chao 1 index, and (**D**) Shannon index of hindgut bacteria.

**Figure 2:**
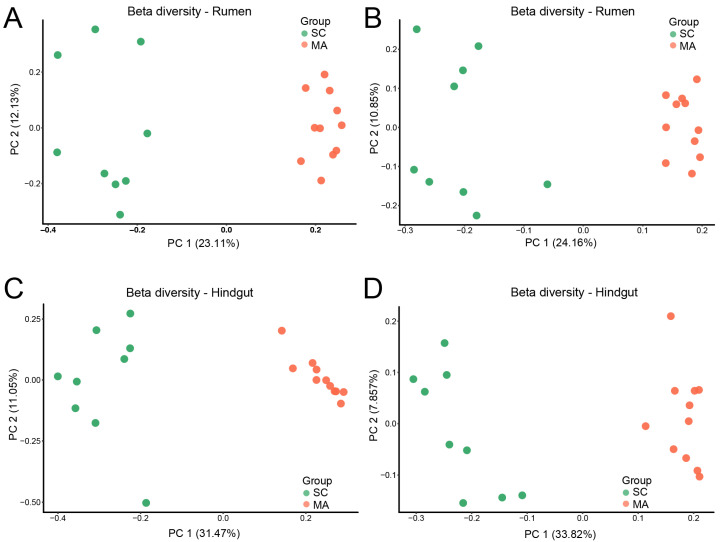
Beta diversity of rumen and hindgut bacteria between high-somatic-cell-counts cows with healthy patterns (SC) and mastitis patterns (MA). Principal variance components analysis (PCoA) with (**A**) Bray–Curtis and (**B**) weighted dissimilarity of rumen bacteria; PCoA with (**C**) Bray–Curtis and (**D**) weighted dissimilarity of hindgut bacteria.

When the alpha-diversity of bacterial communities was compared, the MA cows had a significantly higher richness and evenness (Chao 1 and Shannon indices) than SC cows in the rumen (Figure 1A,B, *p* < 0.01). For hindgut microbiota, there was no significant difference in Chao 1 index (Figure 1C, *p* = 0.70), while a significant difference in the Shannon index was observed (Figure 1D, *p* < 0.01) between SC and MA groups. The PCoA plot based on Bray–Curtis and weighted distance (Figure 2) showed distinct clustering both in rumen and hindgut bacterial communities from SC and MA cows, respectively.

**Figure 3:**
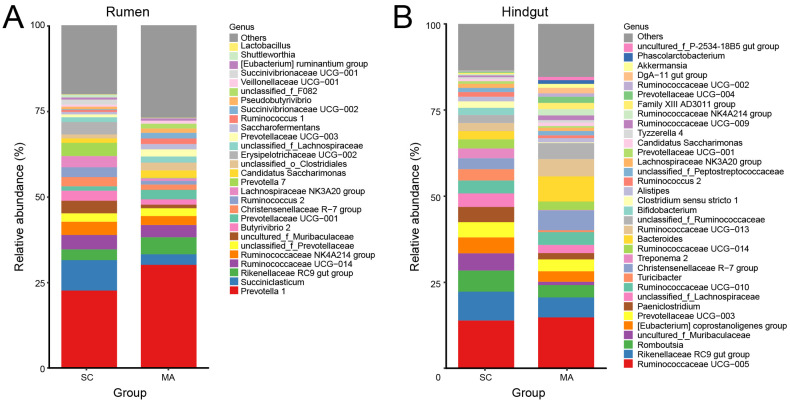
Bar plot of observed bacterial genera with a relative abundance >1% in the (**A**) rumen and (**B**) hindgut from the high-somatic-cell-counts cows with healthy patterns (SC) or with mastitis patterns (MA).

As shown in Figure 3A, twenty-eight rumen bacterial genera were observed with relative abundances greater than 1%. *Prevotella* 1 predominated in all cows, followed by the *Succiniclasticum* and *Rikenellaceae* RC9 gut group. With relative abundances over 0.1%, 51 out of 109 genera showed significantly different abundances (*p* < 0.05) in the rumen between SC and MA cows (Table S4). In the hindgut, there were thirty-four hindgut genera with a relative abundance over 1% (Figure 3B), with *Ruminococcaceae* UCG-005 predominating in all cows, followed by the *Rikenellaceae* RC9 gut group and *Romboutsia*. In the hindgut, 50 out of 91 genera with relative abundances over 0.1% had a significant different abundance (*p* < 0.05) between SC and MA cows (Table S5).

### 3.3. Random Forest Models of Observed Rumen and Hindgut Bacterial Genera

**Figure 4:**
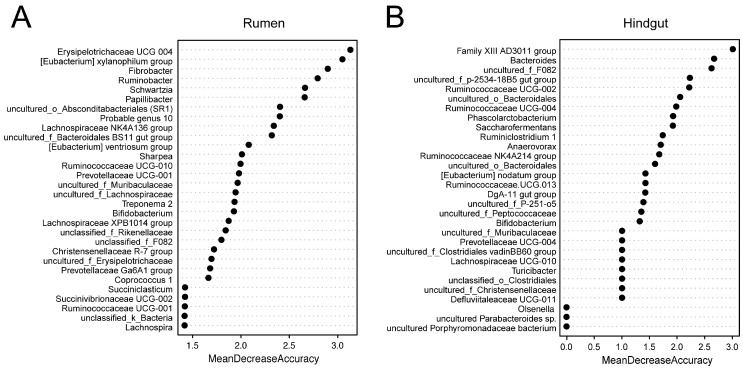
Selected genus in repeated cross-validation of the optimal random forest model.

For the rumen and hindgut microbiome, 25 and 29 genera selected by the random forest modeling approach were explanatory to predict if a cow with high SCC was “true” mastitis with an AUC of 1 in the model we constructed (Figures S1 and S2). The 30 bacterial genera from the rumen and hindgut with the highest MDA are shown in Figure 4, with *Erysipelotrichaceae* UCG 004, the [*Eubacterium*] *xylanophilum* group, and *Fibrobacter* in the rumen; and the Family XIII AD3011 group, *Bacteroides*, and uncultured_f_F082 in the hindgut being the top 3 features, respectively.

The relative abundances of the genera with the top 30 mean decrease in accuracy (MDA) scores from the rumen and hindgut in SC and MA cows are shown in Table 3 and Table 4, respectively. There were 23 genera showing significant differences, and the top 3 genera were more abundant and significantly different (*p* < 0.01) between the rumen of cows from the MA group versus the SC group. In the hindgut, there were 21 genera showing a significant difference between the two groups, and six genera were found to be unique in one group. The top 3 genera Family XIII AD3011 group, *Bacteroides*, and uncultured_f_F082 were observed to be more abundant in the MA group (*p* < 0.01).

### 3.4. Predicting Mastitis Using Rumen Bacteria

Out of 334 dairy cows, 233 dairy cows had SCCs lower than 500 × 10^3^ cells/mL and were classified as healthy, while the remaining 101 dairy cows with SCCs greater than 500 × 10^3^ cells/mL in milk were identified as “mastitis” cases. Relying solely on using the SCC for the identification of mastitis, the incidence rate in this herd was 30.24%.

**Figure 5:**
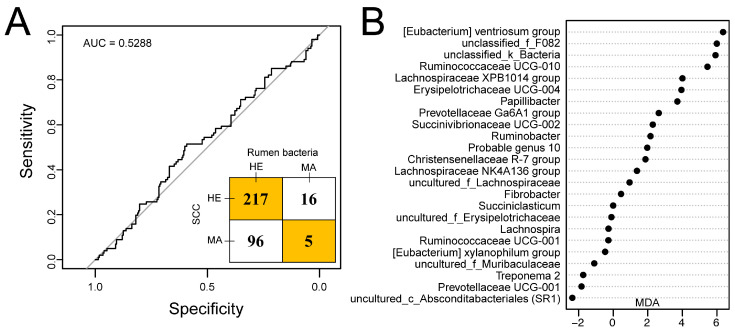
Predicting udder health status outcome using random forest model by using a rumen bacteria dataset of 334 dairy cows [24]. (**A**) Receiver operating characteristic (ROC) curve together with the confusion matrix of the performance of the random forest model with the selected 24 genera. (**B**) 24 genera selected in the random forest model. AUC, area under the curve; HE, cows with somatic cell counts (SCCs) lower than 500 × 10^3^ cells/mL in milk or classified as healthy according to the random forest model; MA, cows with SCCs over 500 × 10^3^ cells/mL in milk or classified as mastitis according to the random forest model; MDA, mean decrease in accuracy.

Twenty-four genera of rumen bacteria were selected by random forest and were observed to have average relative abundances over 0.1%. The receiver operating characteristic (ROC) curve with an AUC of 0.5288 and an inset confusion matrix are shown in Figure 5A. In the herd, a total of 217 out of 233 dairy cows had SCCs lower than 500 × 10^3^ cells/mL in milk and were predicted to be free from mastitis. Surprisingly, there were 96 cows predicted to be healthy according to the random forest model within 101 dairy cows with SCCs greater than 500 × 10^3^ cells/mL in milk. Thus, the predicted incidence rate of mastitis was 6.29%. The MDA score of rumen bacteria selected is shown in Figure 5B with the [*Eubacterium*] *ventriosum* group, unclassified_f_F082, and unclassified_k_*Bacteria* showing the highest MDA score.

## 4. Discussion

### 4.1. Differences Between Cows with High SCC

It is generally accepted that the SCC increases as soon as udder health deteriorates to boost the immune response for the invasion of pathogens [26]. In our study, lower milk yield, lactose percentage, and urea nitrogen in milk of the MA group were observed, indicating that the cows were indeed having mastitis due to the damage of milk-producing epithelial cells and the increase in the permeability of the blood mammary barrier [27,28,29]. Moreover, the urea nitrogen in milk was lower in the MA group while the ruminal ammonia concentration showed no difference between SC and MA groups, which was in accordance with our previous study [17]. The alteration of the VFA in the rumen was observed between SC and MA groups, suggesting an underlying relationship with the mastitis of dairy cows. In our previous study, cows with elevated SCCs showed a higher A:P ratio in the rumen [17], and we observed similar results in this study. Besides the higher A:P ratio, we found a lower concentration of butyrate in MA cows than that in SC cows (Table 2). Butyrate might play a central role in modulating the inflammatory response [30] and may have a protective effect on the blood–milk barrier and reduce the severity of mastitis as observed in mice [15].

By assessing the diversity of microbiota, we observed a higher Shannon index in both the rumen and hindgut of MA cows. It has been suggested that a higher alpha diversity was usually observed in the gut of healthy individuals, while a contradiction of the results was observed [31]. The SC and MA cows clearly differed in their diversity of bacterial communities according to the two metrics comparison, suggesting a distinct bacterial structure between the rumen and hindgut. Usually, cows with mastitis lead to a decreased feed intake, thus influencing the gastrointestinal bacteria [32], and an individual microbiota can maintain its unique composition even after extensive dietary changes, suggesting that the forces controlling ecological homeostasis extend beyond diet [33].

### 4.2. Random Forest Model and Potential Biomarker

Using the random forest modeling approach, we were able to identify rumen and hindgut bacterial communities that accurately (AUC = 1) differentiate cows with “true” mastitis from those with high SCCs. Given the strong discrimination by the random forest model, rumen and hindgut bacterial communities have a promising potential for becoming future biomarkers due to their biological relevance for host health. In the rumen, *Erysipelotrichaceae* UCG-004 was listed as the #1 predictor for “true” mastitis. The members of this bacterial family *Erysipelotrichaceae*, which belongs to the *Firmicutes* phylum, appear to be highly immunogenic [34] and positively correlated with the inflammation of the host via the immunoglobulin or the cytokines [35]. *Schwartzia*, a genus from *Firmicutes*, was reported to utilize only succinic acid [36] and to be more abundant in cows with higher milk production [37]. In our study, *Schwartzia* also showed a higher relative abundance in SC than in MA cows, which might be a result of the lower intake and activity of cows suffering from mastitis. The relative abundance of genus uncultured_o_*Absconditabacteriales* (SR1) was observed to be higher in MA than in the SMC_H group, which was in line with our previous study and indicates that this kind of bacteria might be linked to the deterioration of udder health [17]. Although remaining to be cultivated [38], the family *Absconditabacteriales* was reported to exist in termites [39] and mammalian digestive tracts [40], and also in the healthy human oral microbiome with low abundances generally but several-fold increases in patients with oral diseases [41]. The above results indicate the existence of biomarkers in the rumen and their potential linkage between mastitis of dairy cows.

In the hindgut, three genera from family *Ruminococcaceae* showed higher relative abundances in MA than in SC cows, including *Ruminococcaceae* UCG-002, *Ruminococcaceae* UCG-013, and *Ruminococcaceae* NK4A214. Although the above mechanisms need further investigation, the more abundant genera from *Ruminococcaceae* were also observed in the hindgut [16] and milk [42] in cows with mastitis, indicating a potential linkage to mastitis. It has been reported that bacteria from the family *Ruminococcaceae* can secrete a complex of inflammatory polysaccharides that induce the cytokine secretion and trigger the inflammation in the gut [43]. Moreover, the relative abundance of *Bacteroides* has been observed to be enriched in MA cows. This kind of bacteria can be pathobiont and involved in several diseases such as enteric infection [44]. Besides, bacteria from *Ruminococcaceae* can utilize the mucin, and may directly contribute to the inflammation and breakdown in gut barrier function, known as “leaky gut,” leading to the translocation of certain gut bacteria to the udder and resulting in the mastitis of dairy cows [18,45]. In MA cows, the absence of *Bifidobacterium* was observed in both the rumen and hindgut. It has been well established that *Bifidobacterium* confers positive benefits to the host; thus, depletion of *Bifidobacterium* may weaken the immune system of the host and lead to lower resistance to mastitic pathogens [46,47].

### 4.3. Comparison of SCC and Rumen Bacteria Identification for Mastitis

Despite the studies still being limited, the random forest model has been used for the successful prediction of diarrhea in dairy cows with high accuracy [14], suggesting the possibility for the model application in the discrimination of disease. Interestingly, the predicted incidence rate of mastitis in dairy cattle based on the rumen microbiome with the 24 selected genera was lower than when solely classifying based on SCC (6.29 vs. 30.24%). In those cows with SCCs lower than 500 × 10^3^ cells/mL, the majority of the cows (93.13%) were classified as healthy based on rumen bacteria, suggesting the importance of both SCC and rumen bacteria for detecting mastitis. While 16 cows with SCCs below 500 × 10^3^ cells/mL were identified as mastitis cases, previous studies have not seen a lower limit to the low SCC associated with a reduced incidence of mastitis [48]. Several other studies also demonstrated that cows with low SCCs may have a higher risk for developing mastitis [8]. Although SCCs in milk have been used for the detection of mastitis widely across the globe, all the cows with various SCCs in this herd were in normal lactation and showed no clinical symptoms of mastitis in our study. In cows with SCCs over 500 × 10^3^ cells/mL, most individuals (95.05%) were classified as healthy based on the rumen bacteria, indicating that false predictions may occur. Keeping a reasonable concentration of somatic cells in milk may be acceptable [49]. Therefore, according to the results of the random forest model we developed and the lactation statuses of the herd, cows with high SCCs may not necessarily be mastitic.

Though evaluations of mastitis are commonly based on SCC only and have been carried out for several decades, previous studies suggested that several single parameters such as parity or disease history should be taken into account when mastitis is identified based on SCC data [50]. We suggest, based on our new data, that the combination of rumen bacteria and milk SCC may predict the mastitis more accurately than before. As the rumen is a very dynamic ecosystem, even the new molecular techniques do not give us the whole rumen microbiome picture, and some undetected interactions among the rumen microbiome can exist, which may directly influence the final results [51,52]. The rumen fluid was used in our study, and has fewer microorganisms than rumen digesta, which may need to be collected in future work. On the other hand, we acknowledge the potential bias of the constructed random forest model and the possible variation in accuracy of the bacterial genera in the rumen we selected together with the genera from the hindgut. Therefore, future studies are required to further improve the classifications.

## 5. Conclusions

In conclusion, it may be difficult to distinguish “true” mastitis cases in dairy cattle only according to the milk SCC. Cows with similarly high milk SCCs showed differences in milk performance, rumen fermentation, and rumen and hindgut bacterial communities. Using a random forest modeling approach, we identified specific bacterial genera that may have predicting power to classify “true” mastitis status for cows with high milk SCCs. The full information content to use the rumen microbiome in dairy cows to predict mastitis status requires further attention. Though the full pictures of rumen and hindgut microbiome remain to be further investigated, our findings may improve the knowledge of the microbial communities residing in the rumen and hindgut of dairy cows from mastitis conditions.

## Figures and Tables

**Table 1:** Milk performance characteristics of dairy cows from the subclinical mastitis (SC) and true mastitis (MA) group.

Item	SC ^1^	MA ^2^	SEM	*p*-Value
Parity	1.89	2.18	0.21	0.42
Days in milk	165.67	166.27	5.33	0.88
Somatic cell counts, 10^3^/mL	2892	2169	637.0	0.30
Milk yield, kg/d	21.51^a^	9.82 ^b^	2.08	<0.01
Protein, %	3.80	3.60	0.08	0.12
Fat, %	3.60	4.32	0.16	0.09
Lactose, %	4.22 ^a^	3.26 ^b^	0.21	0.04
Milk urea nitrogen, mg/dL	15.06 ^a^	7.44 ^b^	0.97	<0.01

^1^ SC, high-SCC cows with healthy patterns. ^2^ MA, high-SCC cows with mastitis patterns. ^a,b^ Means within the same row followed by different superscripts differ at *p* < 0.05.

**Table 2:** Observed rumen fermentation parameters of dairy cows from SC and MA groups.

Item	SC ^1^	MA ^2^	SEM	*p*-Value
Rumen pH	6.63	6.56	0.06	0.69
Ammonia nitrogen, mg/dL	6.24	7.11	0.46	0.46
Total volatile fatty acid, mmol/L	85.10	83.96	3.97	0.37
Molar proportion, mmol/100 mmol				
Acetate (A)	64.67 ^b^	70.38 ^a^	0.77	<0.01
Propionate (P)	19.30	17.48	0.45	0.10
Butyrate	11.86 ^a^	9.00 ^b^	0.46	<0.01
Isobutyrate	1.08	0.86	0.07	0.13
Valerate	1.39 ^a^	1.10 ^b^	0.05	0.01
Isovalerate	1.69 ^a^	1.17 ^b^	0.11	0.02
A:P ratio	3.41 ^b^	4.04 ^a^	0.12	0.01

^1^ SC, high-SCC cows with healthy patterns. ^2^ MA, high-SCC cows with mastitis patterns. ^a,b^ Means within the same row followed by different superscripts differ at *p* < 0.05.

**Table 3:** Relative abundance of rumen bacteria with the top 30 mean decrease in accuracy between the two groups.

Item	SC ^1^	MA ^2^	SEM	*p*-Value
*Erysipelotrichaceae* UCG-004	0.07 ^b^	0.30 ^a^	0.04	<0.01
[*Eubacterium*] *xylanophilum* group	0.01 ^b^	0.17 ^a^	0.03	<0.01
*Fibrobacter*	0.04 ^b^	0.33 ^a^	0.05	<0.01
*Ruminobacter*	0.03 ^b^	0.53 ^a^	0.12	<0.01
*Schwartzia*	0.74 ^a^	0.09 ^b^	0.12	<0.01
*Papillibacter*	0.02 ^b^	0.39 ^a^	0.06	<0.01
uncultured_o_*Absconditabacteriales* (SR1)	0.25 ^b^	0.86 ^a^	0.11	<0.01
probable Genus 10	0.07 ^b^	0.36 ^a^	0.05	<0.01
*Lachnospiraceae* NK4A136 group	0.11 ^b^	0.41 ^a^	0.07	<0.01
uncultured_f_*Bacteroidales* BS11 gut group	0.24 ^b^	0.65 ^a^	0.11	0.02
[*Eubacterium*] *ventriosum* group	0.05 ^b^	0.40 ^a^	0.06	<0.01
*Sharpea*	0.17	nd	0.04	-
*Ruminococcaceae* UCG-010	0.13 ^b^	0.61 ^a^	0.08	<0.01
*Prevotellaceae* UCG-001	1.26 ^b^	2.77 ^a^	0.28	<0.01
uncultured_f_*Muribaculaceae*	0.52	0.71	0.13	0.65
uncultured_f_*Lachnospiraceae*	0.19 ^b^	0.45 ^a^	0.05	<0.01
*Treponema* 2	0.24 ^b^	1.03 ^a^	0.15	<0.01
*Bifidobacterium*	0.48 ^a^	0.01 ^b^	0.11	<0.01
*Lachnospiraceae* XPB1014 group	0.55	0.73	0.09	0.15
unclassified_f_*Rikenellaceae*	0.04 ^b^	0.43 ^a^	0.13	<0.01
unclassified_f_F082	0.27 ^b^	1.19 ^a^	0.16	<0.01
*Christensenellaceae* R-7 group	2.69	1.54	0.41	0.54
uncultured_f_*Erysipelotrichaceae*	0.04 ^b^	0.33 ^a^	0.06	<0.01
*Prevotellaceae* Ga6A1 group	0.12 ^b^	0.32 ^a^	0.04	0.01
*Coprococcus* 1	0.13	0.04	0.02	0.25
*Succiniclasticum*	8.91	3.09	1.21	0.15
*Succinivibrionaceae* UCG-002	0.24 ^b^	1.68 ^a^	0.35	0.01
*Ruminococcaceae* UCG-001	0.05	0.25	0.05	0.07
unclassified_k_*Bacteria*	0.04	0.08	0.01	0.02
*Lachnospira*	0.47 ^a^	0.36 ^b^	0.11	0.02

^1^ SC, high-SCC cows with healthy patterns. ^2^ MA, high-SCC cows with mastitis patterns. ^a,b^ Means within the same row followed by different superscripts differ at *p* < 0.05.

**Table 4:** Relative abundances of hindgut bacteria with the top 30 mean decrease in accuracy between the two groups.

Item	SC ^1^	MA ^2^	SEM	*p*-Value
Family XIII AD3011 group	0.36 ^b^	1.79 ^a^	0.19	<0.01
*Bacteroides*	2.38 ^b^	7.29 ^a^	0.70	<0.01
uncultured_f_F082	0.02	0.54	0.07	<0.01
uncultured_f_p-2534-18B5 gut group	nd	0.95	0.25	-
*Ruminococcaceae* UCG-002	0.19 ^b^	0.98 ^a^	0.11	<0.01
uncultured_o_ *Bacteroidales*	0.07 ^b^	0.59 ^a^	0.07	<0.01
*Prevotellaceae* UCG-004	0.34 ^b^	1.80 ^a^	0.24	<0.01
*Phascolarctobacterium*	0.06 ^b^	1.09 ^a^	0.13	<0.01
*Saccharofermentans*	0.01 ^b^	0.23 ^a^	0.03	<0.01
*Ruminiclostridium* 1	0.01 ^b^	0.22 ^a^	0.03	<0.01
*Anaerovorax*	nd	0.28	0.04	-
*Ruminococcaceae* NK4A214 group	0.41 ^b^	1.84 ^a^	0.17	<0.01
uncultured_o_*Bacteroidales*	0.01	0.05	0.02	0.76
[*Eubacterium*] *nodatum* group	0.04 ^b^	0.27 ^a^	0.04	0.01
*Ruminococcaceae* UCG-013	2.37 ^b^	5.00 ^a^	0.44	<0.01
DgA-11 gut group	0.08 ^b^	1.61 ^a^	0.21	<0.01
uncultured_f_P-251-o5	nd	0.25	0.03	-
uncultured_f_*Peptococcaceae*	0.03 ^b^	0.18 ^a^	0.02	<0.01
*Bifidobacterium*	2.06	nd	0.50	-
unclassified_f_*Muribaculaceae*	0.42 ^a^	0.13 ^b^	0.06	0.02
*Prevotellaceae* UCG-004	0.34 ^b^	1.80 ^a^	0.24	<0.01
uncultured_f_*Clostridiales* vadinBB60 group	0.19	0.03	0.03	0.07
*Lachnospiraceae* UCG-010	0.09 ^b^	0.63 ^a^	0.07	<0.01
*Turicibacter*	3.29 ^a^	0.53 ^b^	0.48	<0.01
unclassified_o_*Clostridiales*	0.12 ^b^	0.54 ^a^	0.06	<0.01
uncultured_f_*Christensenellaceae*	nd	0.11	0.02	-
*Defluviitaleaceae* UCG-011	0.08 ^b^	0.17 ^a^	0.02	0.01
*Olsenella*	0.14	0.34	0.07	0.20
uncultured *Parabacteroides* sp.	0.32	nd	0.14	-
uncultured *Porphyromonadaceae* bacterium	0.63 ^a^	0.08 ^b^	0.10	<0.01

^1^ SC, high-SCC cows with healthy patterns. ^2^ MA, high-SCC cows with mastitis patterns. ^a,b^ Means within the same row followed by different superscripts differ at *p* < 0.05.

## Supplementary Materials

The following are available online at https://www.mdpi.com/2076-2607/8/12/2042/s1, Figure S1: ROC curve of the random forest model for predicting udder health with rumen microbiota dataset. Figure S2: ROC curve of the random forest model for predicting udder health with hindgut microbiota dataset. Table S1: Ingredients and chemical composition of the experimental diet. Table S2: Relative abundance of rumen bacteria and SCC records of 334 dairy cows. Table S3: Summary of sequencing results for rumen and hindgut samples. Table S4. Relative abundance of rumen bacteria with relative abundance over 0.1% in at least one group between SC and MA groups. Table S5: Relative abundance of hindgut bacteria with relative abundance over 0.1% in at least one group between SC and MA groups.
